# Heme Uptake by *Leishmania amazonensis* Is Mediated by the Transmembrane Protein LHR1

**DOI:** 10.1371/journal.ppat.1002795

**Published:** 2012-07-12

**Authors:** Chau Huynh, Xiaojing Yuan, Danilo C. Miguel, Rebecca L. Renberg, Olga Protchenko, Caroline C. Philpott, Iqbal Hamza, Norma W. Andrews

**Affiliations:** 1 Department of Cell Biology and Molecular Genetics, University of Maryland, College Park, Maryland, United States of America; 2 Department of Animal and Avian Sciences, University of Maryland, College Park, Maryland, United States of America; 3 Genetics and Metabolism Section, Liver Diseases Branch, NIDDK, National Institutes of Health, Bethesda, Maryland, United States of America; Washington University School of Medicine, United States of America

## Abstract

Trypanosomatid protozoan parasites lack a functional heme biosynthetic pathway, so must acquire heme from the environment to survive. However, the molecular pathway responsible for heme acquisition by these organisms is unknown. Here we show that *L. amazonensis* LHR1, a homolog of the *C. elegans* plasma membrane heme transporter HRG-4, functions in heme transport. Tagged LHR1 localized to the plasma membrane and to endocytic compartments, in both *L. amazonensis* and mammalian cells. Heme deprivation in *L. amazonensis* increased *LHR1* transcript levels, promoted uptake of the fluorescent heme analog ZnMP, and increased the total intracellular heme content of promastigotes. Conversely, deletion of one *LHR1* allele reduced ZnMP uptake and the intracellular heme pool by approximately 50%, indicating that LHR1 is a major heme importer in *L. amazonensis*. Viable parasites with correct replacement of both *LHR1* alleles could not be obtained despite extensive attempts, suggesting that this gene is essential for the survival of promastigotes. Notably, LHR1 expression allowed *Saccharomyces cerevisiae* to import heme from the environment, and rescued growth of a strain deficient in heme biosynthesis. Syntenic genes with high sequence identity to *LHR1* are present in the genomes of several species of *Leishmania* and also *Trypanosoma cruzi* and *Trypanosoma brucei*, indicating that therapeutic agents targeting this transporter could be effective against a broad group of trypanosomatid parasites that cause serious human disease.

## Author Summary

The biological activity of many proteins and enzymes requires heme, a large organic ring containing one iron atom at the center. It has been known for several decades that trypanosomatid protozoa lack several enzymes in the heme biosynthetic pathway. Therefore, unlike mammalian cells that can synthesize heme, these unicellular organisms must acquire heme from the environment. However, the mechanism by which this critical co-factor is transported into trypanosomatid parasites was unknown. In this study we identified LHR1, a trans-membrane protein from *Leishmania amazonensis* that mediates transport of extracellular heme into the parasites. Parasites partially deficient in LHR1 are impaired in heme import, and strains completely deficient do not survive. Genes highly similar to *LHR1* are present in several species of trypanosomatid parasites that cause human disease, identifying this transporter as an important target for the development of anti-parasitic drugs.

## Introduction

*Leishmania* spp. are protozoan parasites from the Trypanosomatidae family. In mammalian hosts *Leishmania* is an obligate intracellular parasite, replicating as amastigotes inside acidic phagolysosomes of macrophages. Disease caused by infection with *Leishmania spp*. has a severe impact on human populations throughout much of the tropics. The clinical manifestations range from self-healing cutaneous lesions to lethal visceralizing disease. In many regions of the world treatment of leishmaniasis still relies on toxic drugs such as pentavalent antimony, which requires high doses and a lengthy course of treatment [1], [2]. Treatment failure is commonly observed with pentavalent antimony [3], and alternative drugs are costly and not widely available in endemic areas. This situation, combined with the recent increase in *Leishmania* infections in urban areas [4], [5], [6], highlights the urgent need for identification of essential parasite molecular pathways that can be targeted by new drugs of lower cost and toxicity.

*Leishmania* species are uniquely dependent on the acquisition of heme from the environment. Heme is a metalloporphyrin that serves as a prosthetic group for proteins that perform critical cellular functions such as oxidative metabolism, oxygen storage and transport, and signal transduction [7]. Unlike mammalian hosts which can synthesize heme [8], *Leishmania* and other trypanosomatid protozoa lack several enzymes in the heme biosynthetic pathway [9], [10] and thus depend on an exogenous supply for survival. *Leishmania amazonensis* acquire exogenous he3me as extracellular promastigotes and also as intracellular amastigotes replicating within macrophages [11]. The existence of a specific transporter or receptor for heme on the *Leishmania* plasma membrane has been speculated, based on reports showing high affinity heme binding to the cell surface of *L. amazonensis* promastigotes [12] and *L. infantum* axenic amastigotes [13], and specific uptake of the porphyrin heme analog MgPPIX in *L. donovani* [14]. However, the nature of the membrane-associated molecule(s) responsible for heme uptake by *Leishmania* has remained unknown. In this study, we identify Leishmania Heme Response-1 (*LHR1*), a *L. amazonensis* gene that shares homology with *HRG-4*, a *C. elegans* gene that encodes a plasma membrane heme importer [15]. We show that *LHR1* transcript levels increase during heme deprivation, and that the LHR1 protein localizes to the plasma membrane and endocytic compartments, promotes heme uptake, and regulates the intracellular pool of heme in the parasites. Our results identify LHR1 as a strong candidate for the elusive transmembrane transporter responsible for heme acquisition from the environment by *Leishmania*.

## Results

### Identification of *LHR1*, a heme-responsive *Leishmania* gene

**Figure 1:**
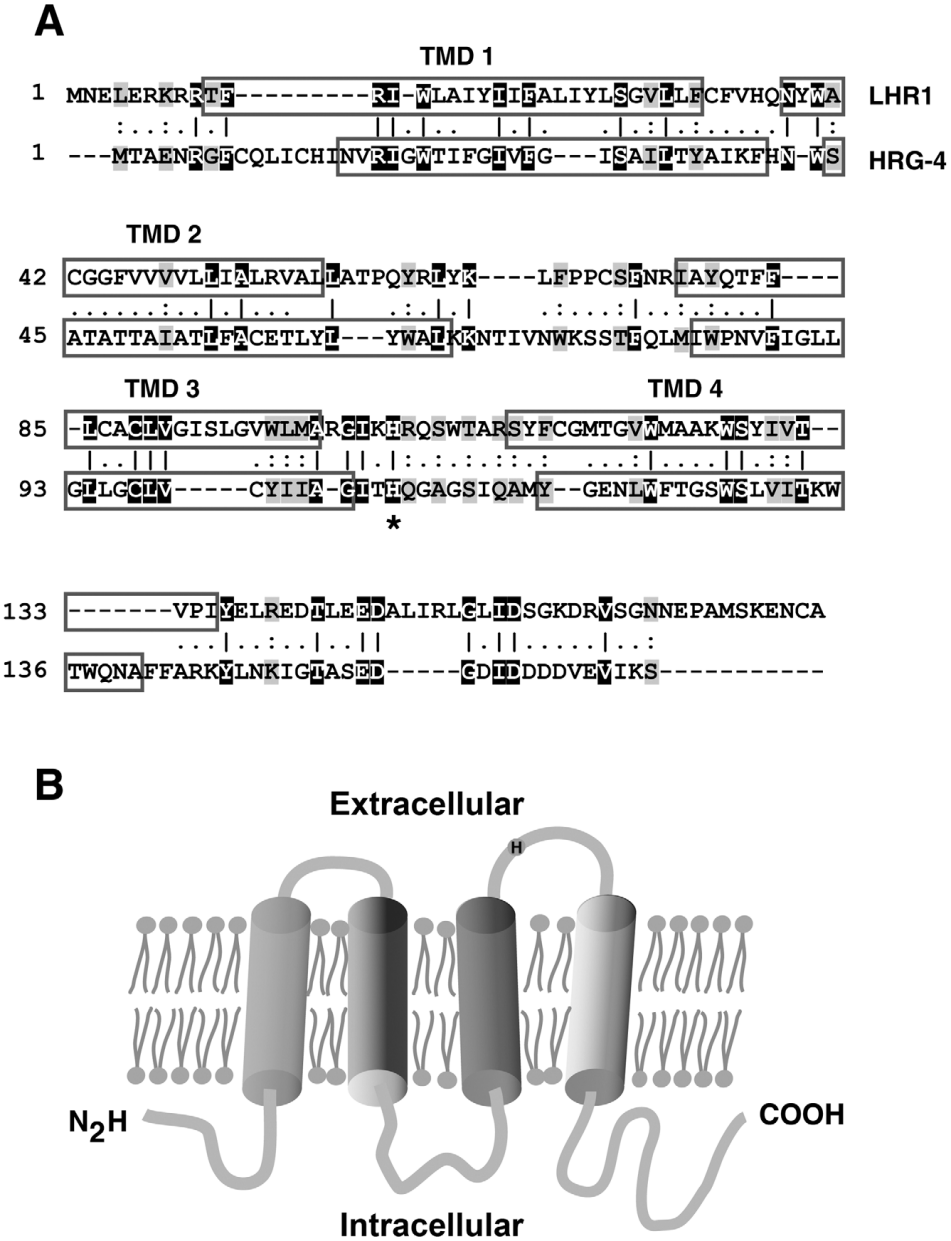
Identification and predicted topology of LHR1, a *Leishmania* transmembrane protein similar to the *C. elegans* heme importer HRG-4. (A) Amino acid alignment of *L. amazonensis* LHR1 and *C. elegans* HRG-4 (ClustalW PAM 250 Lasergene MegAlign software) shows that LHR1 also has four predicted transmembrane domains (TMD 1–4, boxed regions). Identical and conserved amino acids are highlighted in black and gray, respectively, and a histidine shown to be important for heme uptake by CeHRG-4 [16] is indicated by an asterisk. (B) Proposed membrane topology of *Leishmania* LHR1, illustrating the four transmembrane domains and the conserved histidine, based on the CeHRG-4 [15] [16].

The presence of hemoproteins within *Leishmania amazonensis* in the absence of a functional heme biosynthetic pathway [11] suggested the existence of a membrane protein capable of importing heme from the medium. As a strategy to identify this molecule, we searched the TriTryp database (http://tritrypdb.org/tritrypdb/) for genes encoding transmembrane proteins with similarity to CeHRG-4, the prototypical heme transporter from another heme auxotroph, the nematode *C. elegans* [15] (WormBase Gene ID WBGene00009493). In addition to BLAST homology searches, we refined our approach by identifying predicted proteins similar to HRG-4 in size and in the number of putative transmembrane domains. This search strategy identified a single open reading frame of 157 amino acids in chromosome 24, LmjF.24.2230 (*L. major*), LmxM.24.2230 (*L. mexicana*) and LinJ.24.2320 (*L. infantum*). This gene, named *Leishmania* Heme Response-1 (*LHR1*) (Genbank accession number CBZ27556), shares ≈15% identity and ≈45% similarity with *C. elegans* HRG-4 [15] (Figure 1A). We amplified a 525-bp fragment from the *L. amazonensis* genome using nucleotide sequences from the TriTryp database, and amino acid sequence analysis confirmed the presence of the four predicted transmembrane domains also present in CeHRG-4. The predicted transmembrane topology suggests that the N- and C- termini of LHR1 are cytoplasmic, consistent with the proposed topology for CeHRG-4 [15], with extracellular exposure of the conserved histidine shown to be involved in heme uptake [16] (Figure 1B).

**Figure 2:**
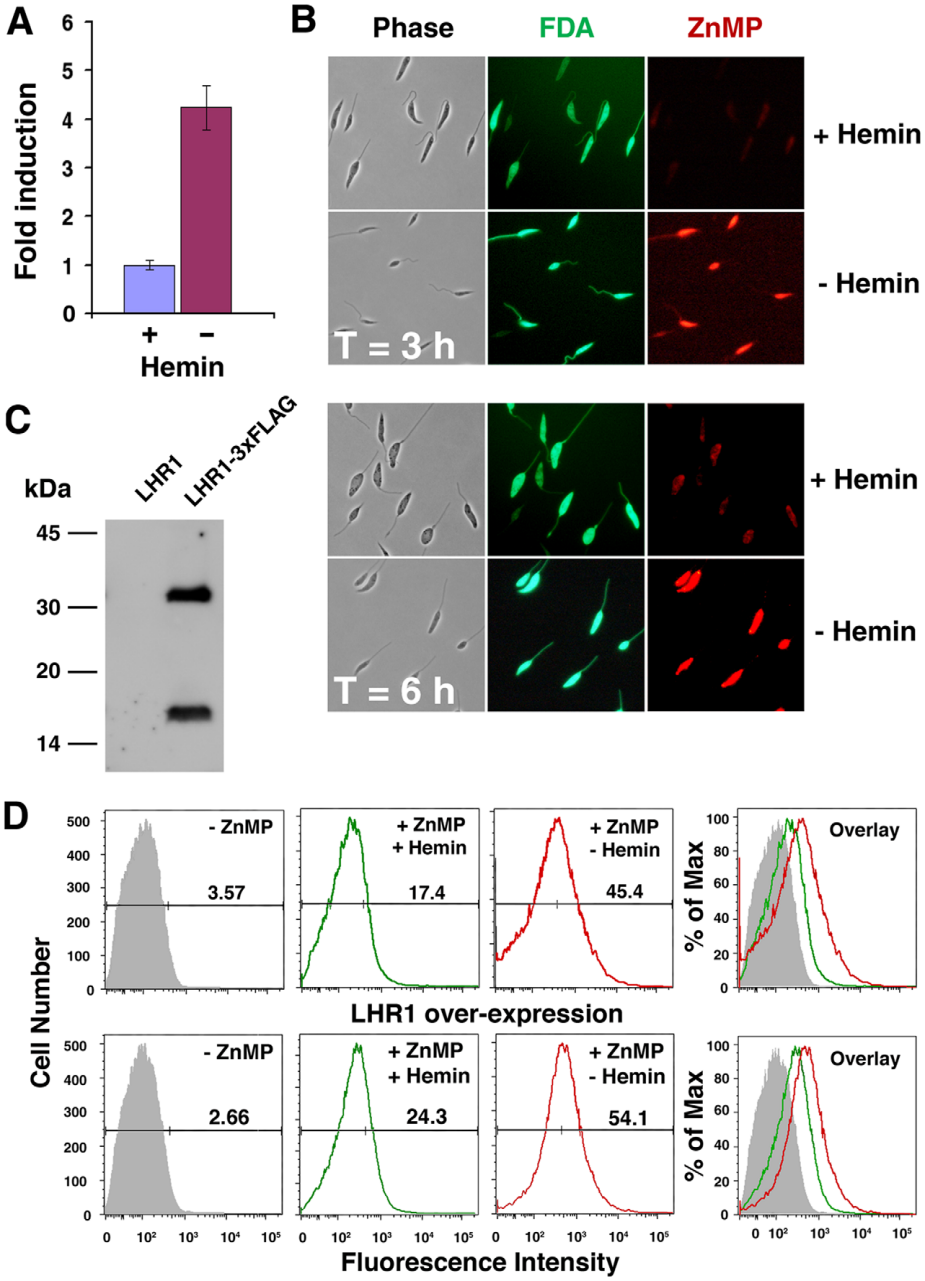
Heme deprivation increases LHR1 expression and promotes uptake of the heme analog ZnMP by *L. amazonensis* promastigotes. (A) qPCR quantification of *LHR1* transcripts relative to the reference gene *GAPDH*, in log phase *L. amazonensis* promastigotes grown in medium with or without heme for 15 h. The results represent the mean+/−standard deviation (SD) of three independent experiments. (B) Confocal fluorescence images of *L. amazonensis* promastigotes cultured for 15 h with or without heme, incubated with ZnMP for 3 or 6 h, and imaged live under identical conditions. ZnMP accumulation within the parasites is shown in red, and the viability dye FDA is shown in green. (C) Immunoblot of extracts of *L. amazonensis* promastigotes expressing *LHR1* tagged or not with the 3xFLAG epitope, and probed with anti-FLAG M2 monoclonal antibodies. (D) Flow cytometry quantification of ZnMP uptake by promastigotes untransfected (top) or transfected with *LHR1* (bottom), after 15 h pre-incubation in the presence or absence of heme, followed by uptake under the same conditions. The numbers indicate the percentage of cells with fluorescence levels above the gate value (vertical line on horizontal bar, determined from measurements on parasites not incubated with ZnMP – grey shaded profile).

Given the relatively low sequence homology between *Leishmania* LHR1 and CeHRG-4, we first investigated whether LHR1 expression was influenced by heme availability. *LHR1* transcript levels were elevated four fold within 15 h when *L. amazonensis* promastigotes were cultured in heme deficient media, compared to heme replete conditions (Figure 2A). This result provided the first indication that LHR1 might be involved in heme homeostasis in *Leishmania*.

### LHR1 promotes uptake of a heme analog and increases the heme content of *Leishmania amazonensis* promastigotes

Given the increase in LHR1 transcripts seen after cultivation in heme-deficient medium (Figure 2A), we investigated whether depriving *L. amazonensis* promastigotes of heme for 15 h led to a subsequent increase in the uptake of fluorescent zinc mesoporphyrin IX (ZnMP), a validated heme analog [17], [18], [19]. Low levels of ZnMP uptake were observed when the parasites were cultured in heme-containing medium, consistent with the low *LHR1* transcript levels observed under these conditions. In contrast, the intracellular ZnMP fluorescence signal increased significantly after promastigotes were pre-incubated for 15 h in heme-deficient medium (Figure 2B). When maintained in regular heme-containing medium and then assayed for ZnMP in the presence or absence of heme no intracellular signal was detected, showing that absence of heme during the 3–6 h period of the assay is not sufficient to promote ZnMP uptake (not shown). This result is consistent with our observations, which indicate that at least 12–15 h of heme deprivation is required to upregulate LHR1. The promastigotes remained fully viable after incubation in the absence of heme, as indicated by the viability indicator fluorescein diacetate (FDA) [20] (Figure 2B). Thus, culture conditions that upregulate LHR1 expression lead to a concomitant increase in heme uptake.

**Figure 3:**
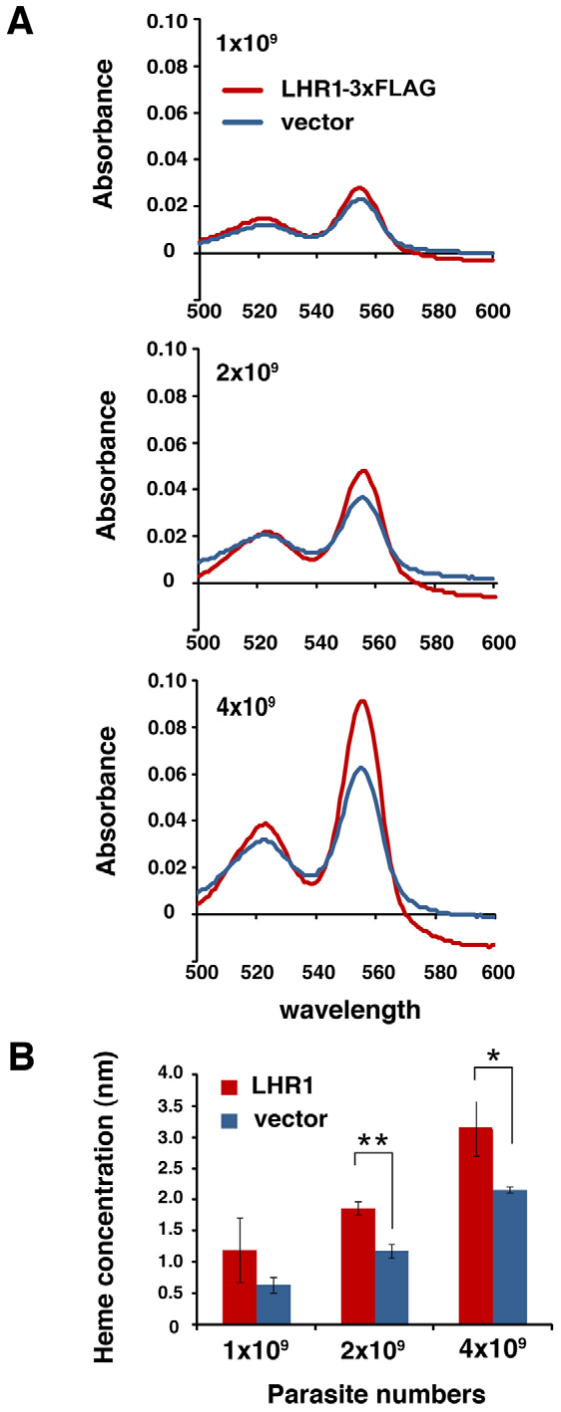
Transfection with LHR1 increases the intracellular heme content of *L. amazonensis* promastigotes. (A) Absorption spectra of the hemochrome content of lysates of increasing numbers of *L. amazonensis* promastigotes transfected with vector alone or with *LHR1-3xFLAG*, and grown in heme-containing medium. (B) Heme concentrations in (A) calculated based on the heme millimolar extinction coefficient of 20.7. The data represents the mean+/−SD of triplicate determinations. p = 0.199 (1×10^9^), ** p = 0.001 (2×10^9^), * p = 0.054 (4×10^9^) (two-tailed Student's t test).

**Figure 4:**
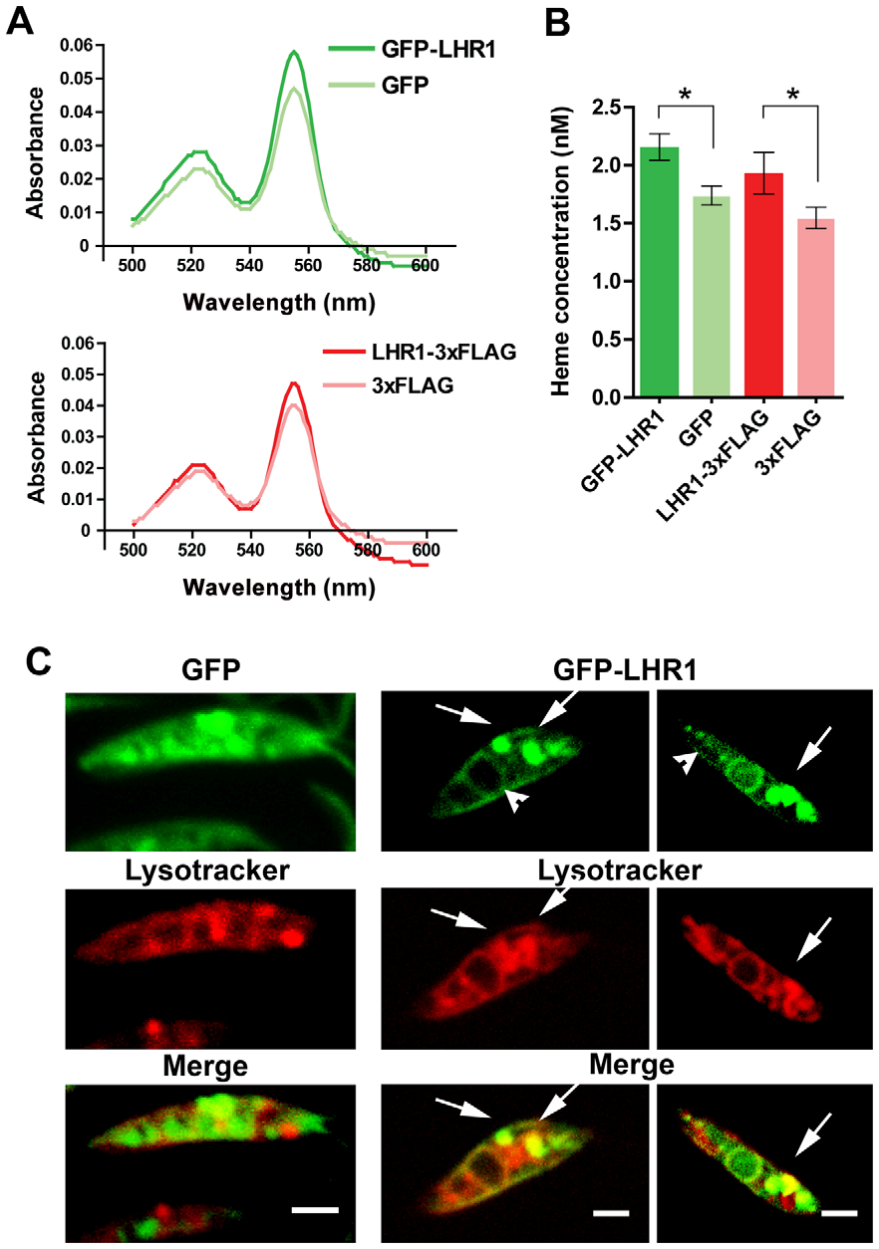
GFP-LHR1 increases the heme content, and localizes to the plasma membrane and acidic endocytic compartments of promastigotes. (A) Absorption spectra of the hemochrome content of lysates of *L. amazonensis* promastigotes (4×10^9^) transfected with vector alone (GFP or 3xFLAG) or *LHR1* (*GFP-LHR1* or *LHR1*-*3xFLAG*), and grown in heme-containing medium. (B) Heme concentrations in (A) calculated based on the heme milimolar extinction coefficient of 20.7. Data are represented as the mean ± standard error of three independent experiments. * p = 0.020 (*GFP-LHR1* vs. GFP), p = 0.031 (*LHR1*-*3xFLAG* vs. *3xFLAG*) (two-tailed Student's t test). (C) Spinning disk confocal microscopy images of live *L. amazonensis* promastigotes transfected with GFP vector alone or *GFP-LHR1* and grown in heme-deficient medium. *GFP-LHR1* is localized on the plasma membrane of promastigotes (arrowheads) and in intracellular compartments that colocalize with lysotracker (arrows). Bars = 3 µm.

To directly examine the role of LHR1 in heme uptake by *L. amazonensis*, promastigote forms were transfected with an episomal expression plasmid carrying *LHR1* tagged with 3xFLAG at the carboxyl terminus. Immunoblot analysis using monoclonal antibodies to the FLAG epitope detected two bands, one migrating at approximately 20 kDa corresponding to the predicted molecular mass of LHR1, and another band at >30 kDa (Figure 2C) that is likely to correspond to oligomers, as previously observed with *C. elegans* HRG-1 [15]. Uptake of the heme analog ZnMP by promastigotes transfected with *LHR1-3xFLAG* was measured by flow cytometry. Compared to untransfected parasites, *LHR1*-transfected promastigotes showed an enhanced fluorescence signal, reflecting an increased ZnMP uptake by the parasites. These values were further increased after pre-incubation of the parasites in heme-deficient medium for 15 h to upregulate LHR1 expression (Figure 2D). Importantly, *LHR1* episomal expression also increased the total intracellular heme content in *L. amazonensis* promastigotes. The increased intracellular heme pool induced by LHR1 expression was observed in several independent experiments, performed with different numbers of promastigotes expressing *LHR1-3xFLAG* (Figure 3A,B), or GFP-LHR1 (Figure 4A, B).

### LHR1 is targeted to the plasma membrane and lysosomal compartments in *Leishmania* and mammalian cells

**Figure 5:**
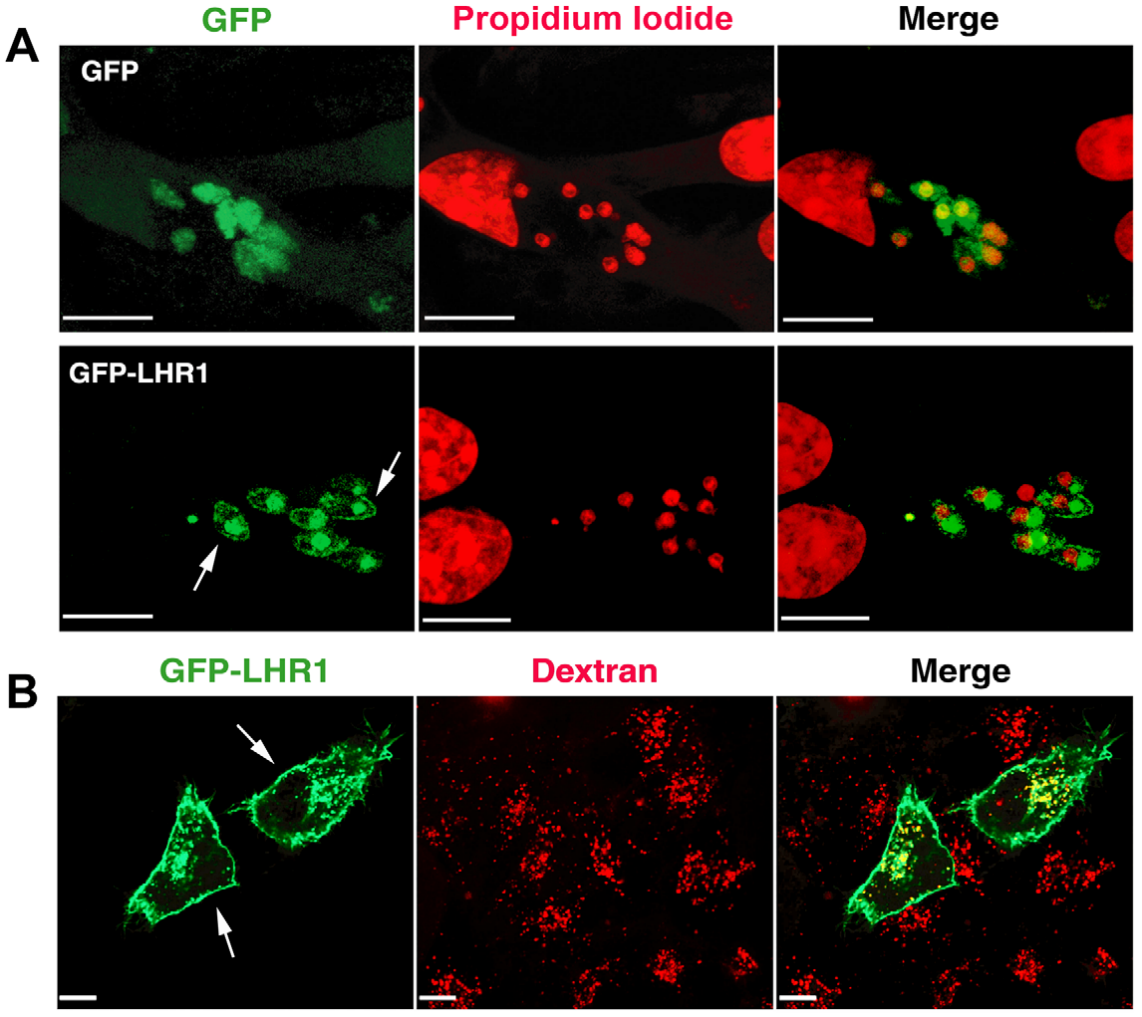
LHR1 localizes to the plasma membrane and late endosomes in intracellular amastigotes and in mammalian cells. (A) Confocal fluorescence images of bone marrow macrophages infected with axenic amastigotes transfected with GFP vector alone or *GFP-LHR1*, and incubated for 48 h. Green, GFP or *GFP-LHR1*; Red, propidium iodide DNA stain. LHR1 is localized on the plasma membrane of intracellular amastigotes (arrows) and in a large intracellular compartment. Bars = 10 µm. (B) Confocal fluorescence imaging of HeLa cells transfected with *GFP-LHR1* and incubated for 1 h with Texas Red dextran followed by a 2 h chase to label lysosomes. *GFP-LHR1* (green) is localized on the plasma membrane (arrows) and in dextran-containing lysosomes (red). Bars = 10 µm.

To determine the sub-cellular localization of LHR1, *L. amazonensis* promastigotes transfected with GFP-LHR1 were cultured overnight in heme-deficient medium and then examined by confocal laser fluorescence microscopy. GFP-LHR1 was detected on the plasma membrane and in acidic intracellular compartments of promastigotes, as indicated by co-localization with lysotraker (Figure 4C). We also examined mouse macrophages infected for 24 h with axenic amastigotes expressing GFP-LHR1. The fluorescent chimeric protein was also localized at the plasma membrane of intracellular amastigotes, and in a large intracellular compartment that is likely to correspond to the megasome, the markedly enlarged lysosomal organelle typical of *L. amazonensis* amastigotes [21]. Parasites expressing GFP alone showed only diffuse cytosolic fluorescence, and no co-localization with lysotracker (Figure 4C, 5A). After ectopic expression in HeLa cells, GFP-LHR1 localized to the plasma membrane and lysosomes, which were identified by co-localization with fluorescent dextran chased for several hours into lysosomes (Figure 5B). Thus, in both *L. amazonensis* and mammalian cells, LHR1 is targeted to the plasma membrane and to late endosomes/lysosomes, two cellular sites from where heme can be transported into the cytosol.

### LHR1 promotes heme uptake and functionally rescues a yeast strain defective in heme biosynthesis

**Figure 6:**
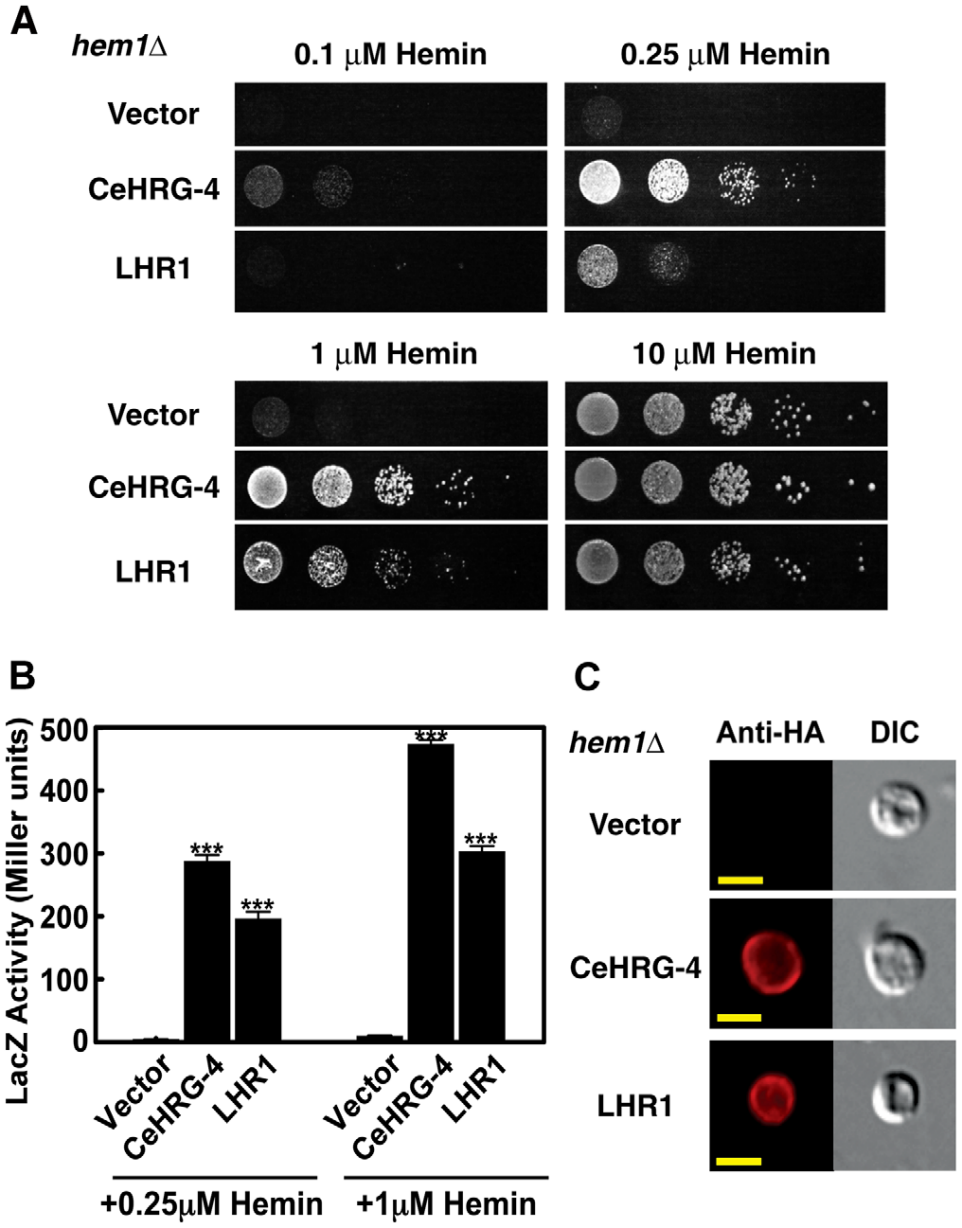
LHR1 rescues heme deficiency phenotypes in *hem1Δ S. cerevisiae*. (A) Spot growth assay of the heme biosynthesis deficient yeast strain *hem1Δ* in heme limiting conditions. *hem1Δ* were transformed with pYes-DEST52 vector alone, *CeHRG-4* or *LHR1*, spotted in serial dilutions on plates supplemented with indicated concentration of hemin, and incubated at 30°C for 3 days. (B) β-galactosidase activity in the *hem1Δ* strain co-transformed with pCYC1-LacZ and empty vector pYes-DEST52, *CeHRG-4* or *LHR1*, determined after cultivation with the indicated concentrations of hemin for 12 h. The results represent the mean+/−standard error of the mean (SEM) from three independent experiments. *** p<0.001 (Student's t test). (C) Epifluorescence images of *hem1Δ* yeast transformed with pYes-DEST52 vector alone, *CeHRG-4-HA* or *LHR1-HA* and subjected to immunofluorescence with anti-HA rabbit antibodies. Bar = 5 µm.

**Figure 7:**
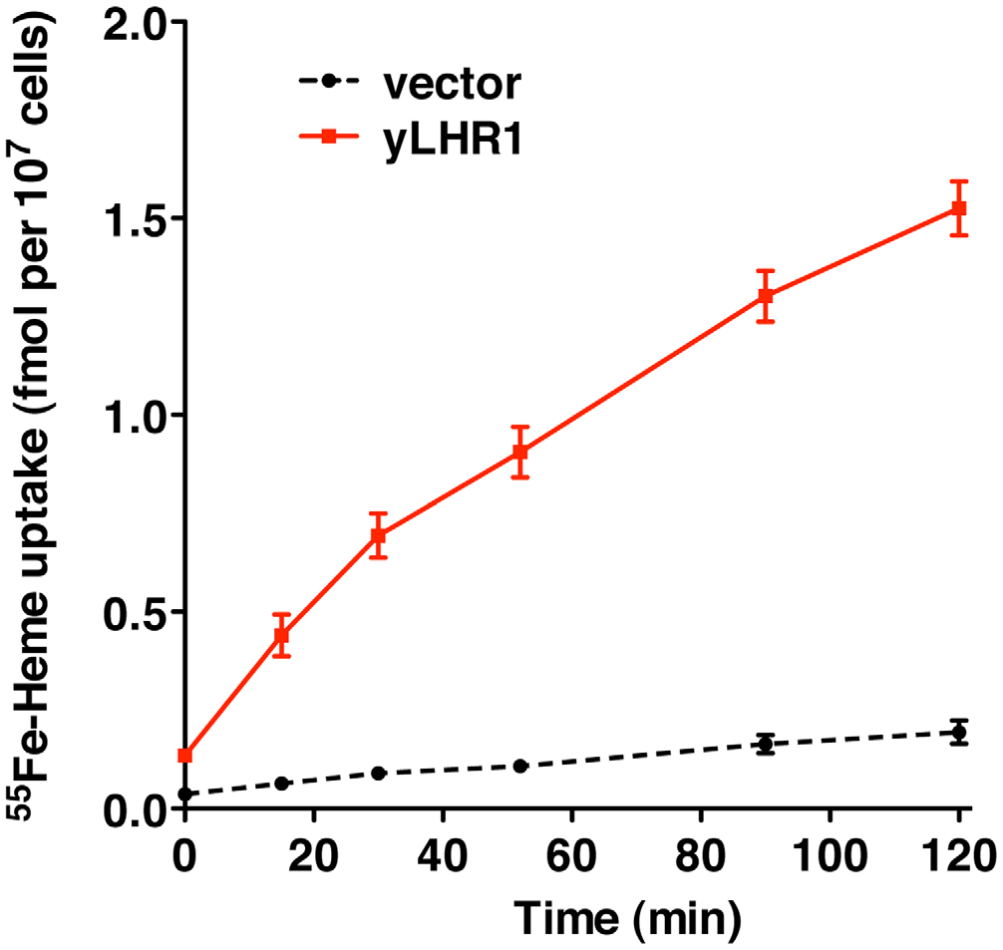
LHR1 promotes heme uptake in *S. cerevisiae*. Wild-type yeast transformed with yeast-optimized LHR1 (yLHR1) or the empty vector (vector) were grown in SC-Ura, 2% raffinose, 0.4% galactose medium and uptake of [^55^Fe] hemin was measured. Assays were performed with [^55^Fe] hemin at 1.2 µM and uptake was measured for the indicated number of minutes at 30°C. Assays were performed in triplicate and the experiment was replicated twice. The data represents the mean+/−SD of triplicate determinations.

To obtain direct evidence for the ability of LHR1 to transport heme across membranes and make it available for essential metabolic reactions in the cytosol, we performed heterologous expression in *S. cerevisiae*. This unicellular eukaryote utilizes exogenous heme very poorly, even when it lacks a heme biosynthetic pathway [22] [16]. The *S. cerevisiae hem1*Δ strain lacks δ-aminolevulinic acid synthase (ALAS), the first enzyme in the heme biosynthesis pathway. To grow, this strain requires supplementation of either δ-aminolevulinic acid (ALA), the product of ALAS, or excess hemin (≥10 µM) in the growth medium [23]. We found that *hem1Δ* requires 40-fold less hemin in the growth medium when transformed with either *LHR1* or the *C. elegans* heme transporter *CeHRG-4* (Figure 6A). We also used yeast assays to measure changes in regulatory intracellular pools of heme promoted by *LHR1*. We found that *hem1Δ* cells expressing a *CYC1::lacZ* promoter-reporter fusion transformed with either *LHR1* or *CeHRG-4* showed more than 30-fold increase in β-galactosidase activity activity (Figure 6B). This is a direct indication of heme transport, since in this system CYC1 and *lacZ* expression depend on Hap1p, a transcription factor that binds heme [24]. Immunofluorescence microscopy showed that LHR1 was also expressed on the plasma membrane of *S. cerevisiae*, consistent with its subcellular localization in promastigotes and HeLa cells (Figure 6C). These results show that plasma membrane-localized LHR1 is capable of conferring to *hem1Δ S. cerevisiae* the ability to import heme from the environment. This was directly demonstrated in kinetic experiments, which showed that LHR1 expression in wild type *S. cerevisiae* promotes incorporation of ^55^Fe-heme from the medium as a function of time (Figure 7).

### Deletion of one *LHR1* allele inhibits uptake of a heme analog and reduces the total heme content of *Leishmania amazonensis* promastigotes

**Figure 8:**
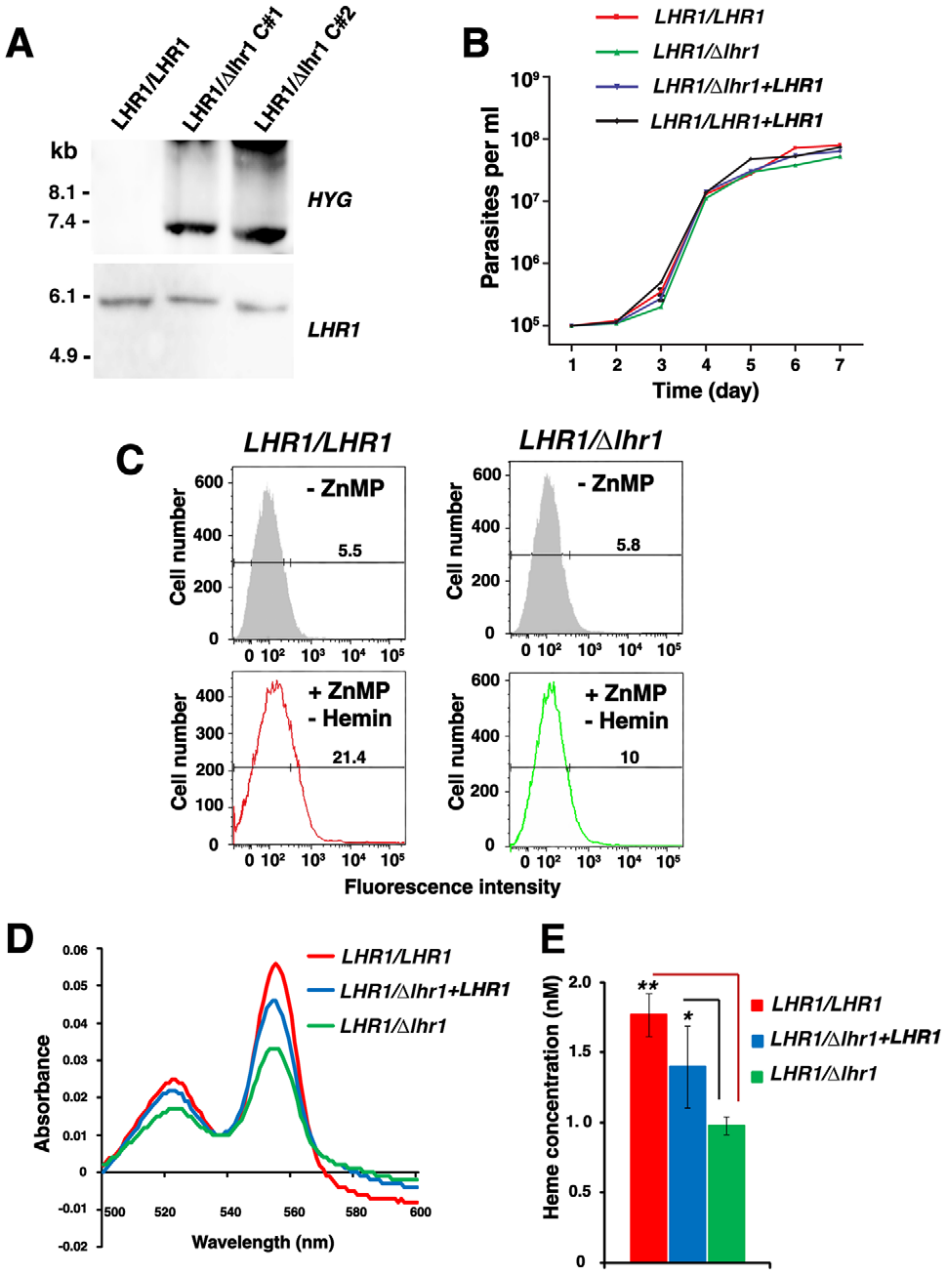
*LHR1* is required for maintaining heme homeostasis in *L. amazonensis*. (A) Southern blot of genomic DNA from wild type (*LHR1/LHR1*) and heterozygous (*LHR1/Δlhr1*) promastigotes digested with *Xho*I and hybridized with *LHR1* or *HYG* ORFs. Two independent hygromycin B resistant clones were used for genomic DNA isolation (*LHR1/Δlhr1* C#1 and C#2). (B) Growth curves of *LHR1/LHR1* and *LHR1/Δlhr1* promastigotes expressing or not episomal *LHR1-3xFLAG* in promastigote growth medium containing hemin. (C) Flow cytometry quantification of ZnMP uptake by *LHR1/LHR1* and *LHR1/Δlhr1* promastigotes kept in heme-deficient medium during the 15 h pre-incubation and the 3 h assay. The numbers indicate the percentage of cells with fluorescence levels above the gate value (vertical line on horizontal bar, determined from measurements on parasites not incubated with ZnMP – grey shaded profile). (D) Absorption spectra of the hemochrome content of lysates of 4×10^9^ *LHR1/LHR1* (red), *LHR1/Δlhr1* expressing LHR1-3xFLAG (blue) or *LHR1/Δlhr1* (green) *L. amazonensis* promastigotes grown in heme-containing medium. (E) Heme concentrations in (D) calculated based on the heme millimolar extinction coefficient of 20.7. The results correspond to the mean+/−SD of triplicate determinations. ** p<0.0004; * p<0.022 (two-tailed Student's *t* test).

To genetically examine the function of LHR1 in heme transport, we generated a *LHR1* mutant in *L. amazonensis* using homologous recombination. The linearized *HYG* gene replacement construct was transfected into promastigotes, and genomic DNA from hygromycin B-resistant and wild type promastigote clones was isolated, digested with *Xho*I and analyzed by Southern blotting. Hybridization with the *LHR1* probe detected a single DNA fragment of 6013 bp in genomic DNA from wild type and two independent hygromycin-resistant clones, as expected based on the presence of restriction sites for *Xho*I in the upstream and downstream genes, but not in *LHR1* and *HYG* coding sequences (Figure 8A). In contrast, hybridization with the *HYG* probe detected a single band of 6,511 bp, consistent with the ≈6,500 bp band expected to be generated by substitution of *LHR1* for *HYG*. These results demonstrated integration of *HYG* marker into the *LHR1* locus, and replacement of a single *LHR1* allele (Figure 8A).

The single knockout (*LHR1/Δlhr1*) mutant strain showed no obvious growth defect when cultivated in regular, hemin-containing medium, with or without transfection with *LHR1-3xFLAG* (Figure 8B). However, following incubation in heme-depleted medium, a condition that upregulates LHR1 expression (Figure 2A), the level of ZnMP uptake over 2 h was reduced by ≈50% in *LHR1/Δlhr1* when compared to wild type (Figure 8C). The total intracellular heme content was also reduced by ≈50% in *LHR1/Δlhr1* promastigotes, a phenotype that was partially restored by transfection of *LHR1/Δlhr1* parasites with the LHR1-3xFLAG episomal plasmid (Figure 8D,E).

Repeated attempts were made to replace the second *LHR1* allele with a *NEO* deletion construct without recovery of viable clones, suggesting that *LHR1* is an essential gene in *L. amazonensis*. When attempts were made to delete the second *LHR1* allele in *LHR1/Δlhr1* parasites transfected with the *LHR1-3xFLAG* plasmid, the only viable promastigotes triple resistant to hygromycin, neomycin and nourseothricin (resistance conferred by the episomal LHR1-3xFLAG plasmid) that were recovered still had one endogenous LHR1 copy (data not shown). These results suggest that the levels of heme transport conferred by episomal expression of LHR1-3xFLAG are not sufficient to sustain promastigote growth, even when an abundant source of heme is provided in the culture medium. This observation is consistent with the incomplete rescue of the heme uptake and homeostasis phenotypes of *LHR1/Δlhr1 L. amazonensis* by LHR1-3xFLAG expression (Figure 8). Taken together, these results strongly suggest that LHR1 accounts for the majority of the heme transport activity of *L. amazonensis*.

## Discussion

The existence of an essential pathway for acquisition of exogenous heme in *Leishmania* and other trypanosomatid protozoa was proposed decades ago [9], when it became clear that these organisms lack several enzymes of the heme biosynthetic pathway [10]. However, the molecule(s) responsible for this critical activity remained unknown. In this work we identify *LHR1*, a *Leishmania* gene upregulated under heme-deficient conditions that encodes a membrane protein able to promote heme uptake from the environment. Transfection of *Leishmania* with LHR1 promotes uptake of a heme analog and increases the total intracellular heme concentration in the parasites. A *L. amazonensis* single-allele *LHR1* knockout strain shows reduced uptake of a heme analog and has a significantly smaller intracellular heme pool. Viable parasites lacking both chromosomal copies of *LHR1* could not be isolated even when carrying episomal *LHR1*, suggesting that LHR1 performs a critical function that depends on levels of expression not achieved with the tagged LHR1-3xFLAG. Importantly, *LHR1* functionally complemented a yeast strain deficient in heme biosynthesis, in both growth and heme-dependent gene expression assays. These results strongly support a role of LHR1 as a transporter and not a receptor for heme, because yeast cells lack a efficient heme import machinery [22] [16]. The efficiency of heme uptake from the environment may vary among *Leishmania* species, since Campos-Salinas *et al.* reported faster incorporation of Mg-PPIX by *L. donovani* promastigotes [14] than what we observed with *L. amazonensis*. Future studies may provide evidence for the intriguing possibility that LHR1 is differentially expressed in visceral strains of *Leishmania*, a property that might be associated with their increased virulence and capacity to proliferate in internal organs.

*LHR1* was identified based on its partial sequence identity and similarity to *HRG-4*, a gene encoding a plasma membrane heme transporter in the nematode *C. elegans*. *HRG-4* was identified in a genetic screen designed to take advantage of the heme auxotrophy of *C. elegans* to identify heme-responsive genes [15]. LHR1 and CeHRG-4 [15] [16] have a similar molecular mass (∼20 kDa), and four predicted transmembrane domains. One intriguing difference observed between CeHRG-4 and LHR1 is their subcellular localization. While HRG-4 is localized primarily on the plasma membrane, GFP-tagged LHR1 was detected on the plasma membrane and on endocytic compartments of *L. amazonensis*. In mammalian cells, GFP-LHR1 was also targeted to the plasma membrane and lysosomes, strongly suggesting that the large intracellular compartments containing LHR1 in *L. amazonensis* correspond to parasite lysosomal compartments. A morphometric and cytochemical study in *L. amazonensis* showed that during differentiation of promastigotes into amastigotes, the lysosomal vacuoles of promastigotes become a megasome, a very large compartments that can comprise up to ∼5% of the total cell volume [21]. This stage-specific lysosomal pattern is very consistent with the intracellular localization of GFP-LHR1 in our study. In addition to the plasma membrane, GFP-LHR1 was observed in several intracellular vesicles in promastigotes and in one very large compartment in intracellular amastigotes.

The dual localization of LHR1 on the plasma membrane and on lysosomes raises interesting questions about the cellular site where heme is translocated into the cytosol. In yeast, LHR1 was targeted to the plasma membrane and promoted functional complementation of a strain incapable of synthesizing heme. However, earlier studies in *Leishmania donovani* showed that hemoglobin is internalized and degraded within parasite lysosomes [25], releasing heme that can then be transported into the cytosol to promote parasite growth. Interestingly, exogenously added hemin rescued the growth of a *L. donovani* strain defective in endocytic transport into lysosomes, indicating that heme translocation across the membrane can occur at both locations – the plasma membrane and the parasite lysosome [26]. The ATP-binding cassette protein LABCG5 was also recently proposed to mediate the salvage of heme released after lysosomal degradation of internalized hemoglobin in *L. donovani*. This intracellular process for heme salvage from degraded hemoglobin was proposed to be distinct from the pathway directly promoting porphyrin transport into the parasites [14]. Additional studies should determine if LHR1 can also mediate the uptake of heme released from hemoglobin inside parasite lysosomes, or if it's primary role is to transport heme from the environment directly across the plasma membrane.

LHR1 null strains could not be generated despite extensive attempts, suggesting that this transporter is essential for the survival of promastigote forms of *L. amazonensis*. Episomal expression of LHR1 increased the intracellular heme concentration of wild type and single knockout *L. amazonensis* promastigotes, but was not sufficient to allow recovery of viable parasites lacking both copies of the gene. This finding is likely related to the fact that episomal LHR1 expression failed to restore the intracellular heme concentration to the same levels observed in wild type parasites. Dysregulated expression and incomplete functional complementation is a frequent observation after episomal or integrated gene expression in *Leishmania* [27], [28], [29], [30]. Incomplete restoration of heme acquisition in *LHR1* double knockout parasites may result in the impairment of critical, essential roles played by hemoproteins in the parasites. For example, LFR1, the recently identified NADPH-dependent ferric iron reductase from *L. amazonensis*, contains a bis-heme motif that is essential for activity and required to allow iron acquisition through the ferrous iron transporter LIT1 [30]. Thus, deleting both copies of *LHR1* may severely affect not only heme uptake, but also the ferrous iron acquisition process.

Searches of the TriTryp database indicate that highly syntenic, close homologs of *L. amazonensis LHR1* (LmxM.24.2230) are present in the additional *Leishmania* species *L. major* (LmjF24.2230), *L. braziliensis* (LbrM.24.2310) and *L. infantum* (LinJ.24.2320), and in the *Trypanosoma* species *T. brucei* (Tb427.08.6010, Tb927.8.6010), *T. brucei gambiense* (Tbg972.8.6030), *T. congolense* (TclL3000.8.5780), and *T. cruzi* (Tc00.1047053511071.190). These trypanosomatid species are the causative agents of serious infectious diseases in humans (*L. infantum*, visceral leishmaniasis; *T. brucei gambiense*, sleeping sickness; *T. cruzi*, Chagas' disease) or in livestock (*T. congolense and T. brucei brucei*, cattle Nagana). Given that the human genome does not include putative orthologs of *CeHRG-4* and *LHR1* [15], our study suggests that LHR1 may represent a promising target for the development of new therapeutic drugs with a potentially broad impact in human health and quality of life.

## Materials and Methods

### Parasite culture

The *L. amazonensis* IFLA/BR/67/PH8 strain was provided by Dr. David Sacks (Laboratory of Parasitic Diseases, NIAID, NIH). Promastigotes were cultured at 26°C in promastigote growth medium: M199 (Gibco BRL) pH 7.4 supplemented with 10% heat-inactivated FBS, 5% penicillin-streptomycin, 0.1% hemin (25 mg/ml in 50% triethanolamine), 10 mM adenine (pH 7.5) and 5 mM L-Glutamine, as previously described [30]. Heme-depleted FBS was generated by treating heat inactivated FBS with 10 mM ascorbic acid for 4 h, followed by dialysis in PBS overnight and filter-sterilization. Heme depletion was verified by measuring the optical absorbance at 405 nm [31]. Parasite viability was assessed by fluorescent microscopy using fluorescein diacetate (FDA; Invitrogen) in combination with Propidium Iodide (PI; Sigma-Aldrich), as described in [20].

### Identification of LHR1, plasmid construction and expression

A single open reading frame, LmjF.24.2230 (*L. major*), LmxM.24.2230 (*L. mexicana*), or LinJ.24.2320 (*L. infantum*) was identified through BLAST homology searches of the *Leishmania* database (TritrypDB). Forward (5′GGATCCATGAACGAGTTGGAGCGC) and reverse (5′GGATCCCTATGCACAGTTCTCC-3′) primers (added *Bam*HI sites underlined) were used to amplify the 525 base pair ORF from genomic DNA of *L. amazonensis*. The PCR product was cloned into the pCR2.1-TOPO (Invitrogen) to generate a *pCR-LHR1* plasmid, and the coding sequence was confirmed by sequencing. To construct a *GFP-LHR1* gene fusion, pCR2.1-*LHR1* digested with BamH1 was cloned into pXGGFP2+ (courtesy of Dr. S. Beverley, Washington University), which drives expression in *Leishmania* of proteins fused to GFP at the N-terminus [32] to yield the *pXG-GFP+LHR1* plasmid. After transfection, clones resistant to G418 (50 µg/ml) were isolated. To generate LHR1 tagged with 3xFLAG at the carboxyl-terminus, the primers 5′GGATCCACCATGAACGAGCGCAAGCG (forward) and 5′GGATCCCTA*TCGCGAT*GCACAGTTCTCCTTTGAC (reverse) (*Bam*HI sites underlined, *Nru*I site in italics) were used to amplify the *LHR1* ORF from pCR2.1-LHR1. The modified *LHR1* ORF containing a *Nru*I site before the stop codon and flanked by BamHI restriction sites was cloned into pCR2.1 using TOPO PCR Cloning kit (Invitrogen) to generate plasmid *pCR2.1-LHR1*(*Nru*l-stop). A *Nru*I excised fragment of the 3xFLAG epitope tag (Sigma-Aldrich) was cloned into *Nru*I digested pCR2.1-*LHR1*(*Nru*l-stop) to generate pCR2.1-*LHR1*-3xFLAG. pCR2.1-*LHR1*-3xFLAG was digested with *Bam*HI and cloned into pXG-SAT (courtesy of Dr. S. Beverley, Washington University) to yield the pXGSAT-*LHR1*-3xFLAG plasmid. After transfection and isolation of clones resistant to 50 µg/ml nourseothricin, LHR1-3xFLAG expression was detected by immunoblot [30]. Total protein extracts (50 µg) from wild type promastigotes expressing either LHR1 or LHR1-3xFLAG were separated on 15% SDS-PAGE, transferred to Immobilon membrane, blocked in 3% nonfat dry milk in TBS (50 mM Tris, 0.138 M NaCl, 2.7 mM KCl, pH8) followed by detection with the mouse monoclonal antibody M2 that recognizes the FLAG epitope (Sigma-Aldrich).

The mammalian expression plasmid *GFP-LHR1* was generated by amplifying GFP-*LHR1* from *pXG-GFP2+-LHR1* plasmid described above, with primers annealing to the start codon of GFP (forward) and to the stop codon of *LHR1* (reverse). The resulting *GFP-LHR1* fragment was cloned into pCR2.1-TOPO to create pCR2.1-*Kpn*I-GFP-*LHR1*-*Xho*I, double digested with *Kpn*I *Xho*I, and the GFP-LHR1 fragment cloned into the *Kpn*I and *Xho*I sites of pShuttle-CMV [32] to yield *pShuttleCMV-GFP-LHR1*.

### LHR1 gene deletion

Gene deletion constructs containing *hygromycin* B *phosphotransferase* (*HYG*) or *neomycin phosphotransferase* (*NEO*) were based on the expression vectors pXG-HYG and pXG-NEO (courtesy of Dr. S. Beverley, Washington University, St. Louis, MO). A 1,000-bp flanking sequence upstream of *L. amazonensis LHR1* was amplified using primers: 5′-GTTGGGCGACTTGTACGG-3′ UPLHR1 (forward) and 5′-GGATCCCGGGTCAACCAAATGCGGAAC-3′ UPREVLHR1 (reverse). A 2,500-bp flanking sequence downstream of *LHR1* was amplified using primers: 5′-GGATCCCGGGCTTGGCCTCATTGATTC-3′ DOLHR1 (forward) and 5′-CCTGTGAAGATGTTCC-3′ DOREVLHR1 (reverse). The upstream and downstream sequences were cloned into pCR2.1-TOPO vector (Invitrogen) creating the plasmid pCR2.1-UP-LHR1 and pCR2.1-DOWN-LHR1. The upstream region was excised from pCR2.1-UP-LHR1 plasmid with *Bam*HI and cloned into pCR2.1-DOWN-LHR1 linearized with *Bam*HI. The resulting plasmid pCR2.1-UP-LHR1-DOWN contained two *Sma*I sites at the junction of the upstream and downstream sequences. To generate the deletion constructs, the *HYG* and *NEO* ORFs were amplified using primers: 5′-GTTCCGCATTTGGTTGGATGAAAAAGCCTGAAC-3′ HYG5primeUTR of LHR1 (HYG forward) 5′-TCAATGAGGCCAAGCCCTATTCCTTTGCCCT-3′ HYG3primeUTR of LHR1 (HYG reverse) and 5′-GTTCCGCATTTGGTTGGATGGGATCGGCCATTG-3′ NEO5primeUTR of LHR1 (NEO forward) 5′-TCAATGAGGCCAAGCCTCAGAAGAACTCGTCAA-3′ NEO3primeUTR of LHR1 (NEO reverse) (underlined sequences corresponding to the *Sma*I site junctions of pCR-UP-LHR1-DOWN) from the pXG-based vectors. Fusion of the ends of amplified *HYG* and *NEO* ORFs to the homologous ends of pCR2.1-UP-LHR1-DOWN linearized with *Sma*I was carried out using Clontech In-Fusion PCR Cloning according to the manufacturer's instructions. DNA fragments from the gene deletion constructs were released from the vector backbone with *Bam*HI and *Xho*I and gel-purified.

Transfections of *L. amazonensis* promastigotes were performed as previously described [30]. To create a *Δlhr1* single knockout strain, the region containing the *LHR1* gene was replaced with the hygromycin B phosphotransferase gene (*HYG*), and clones resistant to hygromycin B (100 µg/ml) were isolated. Southern blots were performed to determine integration of the HYG marker at the *LHR1* locus. Genomic DNA was isolated in TELT lysis buffer as described [33], digested with *Xho*I overnight, separated on 0.8% agarose and transferred to a nylon membrane. Blots were blocked and hybridized to digoxigenin (DIG) labeled PCR probes according to the manufacturer's protocol (Roche). The DIG labeled probes for *HYG* and *LHR1* were generated by PCR amplification with primers: HYG: 5′- ATGAAAAAGCCTGAAC-3′ (forward) and 5′- CTATTCCTTTGCCCT-3′ (reverse); LHR1: 5′GGATCCACCATGAACGAGCGCAAGCG (forward) and 5′ GGATCCCTATCGCGATGCACAGTTCTCCTTTGAC (reverse), using the manufacturer's protocol (Roche). The *LHR1/Δlhr1* single knockout strain was cultured in promastigote growth medium supplemented with 100 µg/ml hygromycin B. Transfection of *LHR1/Δlhr1* with the LHR1-3xFLAG episomal expression plasmid was carried out as described above. The *LHR1* gene deletion procedure was repeated using the *NEO* construct with or without prior transfection with LHR1-3xFLAG.

### Fluorescence microscopy

Transfected *L. amazonensis* promastigote clones expressing GFP-LHR1 were selected in 50 µg/ml G418. To visualize LHR1, log phase promastigotes expressing GFP-LHR1 were imaged live on an UltraView Vox spinning disk confocal system (Perkin Elmer) equipped with an electron multiplier CCD camera (C9100-50; Hamamatsu). Images were acquired and processed using the Volocity software suite (PerkinElmer). For colocalization experiments, parasites were incubated with 1 µM Lysotracker red (InVitrogen) for 20 min at room temperature in serum-free M199 and washed twice before imaging. For LHR1 localization in the intracellular amastigote forms, bone marrow macrophages (prepared from C57BL/6 mice, as described in (25)) infected with *L. amazonensis* axenic amastigotes (differentiated from promastigotes transfected with the GFP vector, GFP-LHR1) were fixed with 2% PFA and treated with 0.1 mg/ml RNase A for 1 h. Samples were washed three times with PBS, stained for 1 min with 50 µg/ml PI, followed by three washes with PBS. Coverslips were mounted in ProLong (Molecular Probes, Invitrogen) and imaged on a confocal microscope (Leica TCS SP5 X) using the application suite software (Leica), followed by image processing with the Volocity software suite.

HeLa CCL-2 cells (HeLa 229) were seeded on 35-mm MatTek dishes (2.0 ml of 0.75×10^5^ cells/ml) 24 h before experiments in DMEM 10% FBS and 1% penicillin/streptomycin, and incubated at 37°C and 5% CO_2_. The media was replaced with 250 µl Opti-MEM I reduced serum and cells were transfected with Lipofectamine and 1 mg pShuttleCMV-GFP-LHR1, according to the manufacturer's instructions (Invitrogen). To label lysosomes, 0.5 mg/ml Texas Red dextran (MW 10,000, Sigma-Aldrich) was added to cells followed by incubation at 37°C for 1 h, several PBS washes and a 2 h chase at 37°C, as previously described [34]. For live imaging, dishes were placed in an environmental chamber (LiveCell System; Pathology Devices, Inc.) at 37°C with 5% CO_2_ attached to an UltraView VoX spinning-disk confocal system (PerkinElmer) equipped with a CCD camera (C9100-50; Hamamatsu) and processed using Volocity software suite (PerkinElmer).

For imaging in *S. cerevisiae*, yeast transformants were cultivated to mid-log phase in 2% w/v raffinose SC (-Ura) medium supplemented with 0.4% w/v galactose and 250 µM ALA, and fixed with 4% formaldehyde for 1 h at room temperature. Immunofluorescence microscopy was performed as described elsewhere [35], and images were acquired using a DMIRE2 epifluorescence microscope (Leica) connected to a Retiga 1300 cooled Mono 12-bit camera.

### Quantitative real time PCR

Log phase growth wild-type *L. amazonensis* promastigotes were washed twice with PBS, resuspended at 10^6^ parasites/ml in promastigote medium without hemin and 20% heme-depleted FBS, or regular promastigote medium with hemin and 20% untreated FBS. After 15 h at 26°C, a total of 10^8^ promastigotes were collected. Three independent samples were used to isolate total RNA using Qiagen RNAeasy kit (Qiagen) according to the manufacturer's instructions. cDNA synthesis was carried out using 1 µg of total RNA and Superscript II Reverse Transcriptase (Invitrogen) according to the manufacturer's protocol. To quantify *LHR1* transcript levels in each sample, 1 µl of the synthesized cDNA was amplified using *LHR1* specific primers: sense 5′-CGCTCGTACTTTTGTGGA-3′ and antisense 5′-CCTGAATCAATGAGGCCAAG-3′, and GAPDH specific primers: sense 5′-GAAGTACACGACCTTCTTC and antisense 5′-CGCTGATCACGACCTTCTTC as the reference gene. Quantitative real time PCR was performed using a BioRad iCyler iQ Real-Time PCR System (BioRad Laboratories) using the SYBR green fluorophore, according to the manufacturer's instructions. All reactions were performed in triplicate, and no template DNA was included in each experiment as a negative control. The cycle threshold (Ct) value was determined, and the fold induction of *LHR1* transcripts was calculated using the 2^−*ΔΔ*Ct^ method [36].

### Measurement of total intracellular heme concentration

Heme (iron protoporphyrin IX) concentration was determined using the pyridine hemochrome method [37]. Log phase growth promastigotes cultivated in regular promastigote growth medium were collected by centrifugation, counted, washed once with PBS, resuspended in 1 ml of 1 mM Tris-HCl pH 8.0, and sonicated for 2 min in an ice water bath using a Branson digital sonifier at 30% power setting, in pulses of 5 seconds intercalated with 5 s of cooling. Aliquots of 840 µl were transferred to 13×100 mm glass tubes, 100 µl of 1 N NaOH was added followed by vortexing, and after 2 min 200 µl of pyridine solution (Sigma-Aldrich) was added to each sample followed by vortexing. Samples were then transferred to a 1 ml cuvette and a baseline absorbance spectrum was obtained. A few sodium dithionite crystals (2–3 mg) were added to the sample, and the reduced hemochrome absorbance spectrum between 500 and 600 nm was acquired after 1 min. Heme concentrations were calculated based on the millimolar extinction coefficient of 20.7, for the difference in absorption between the spectrum peak at 557 nm and the valley at 541 nm.

### ZnMP uptake and quantitation

Zn(II) Mesoporphyrin IX (ZnMP) (Frontier Scientific) was dissolved in DMSO at 10 mM, and the uptake assay was performed as described [14], with modifications. Wild type and LHR1-3xFLAG-expressing *L. amazonensis* promastigotes (10^7^ parasites per ml) were cultured in regular or heme-deficient medium (M199, 10% heme-depleted FBS, 1% Pen/Step) for 15 h. 2×10^9^ parasites were collected by centrifugation, washed once with PBS and divided equally into regular promastigote growth medium or heme-deficient medium containing 10 µM ZnMP for 3 or 6 h. The ZnMP uptake reaction was stopped by adding an equal volume of ice-cold 5% BSA in PBS, parasites was collected by centrifugation, washed, and the fluorescence intensity of ZnMP accumulation in a total of 3×10^4^ parasites was measured by flow cytometry (BD FACSCanto, excitation at 488 nm and emission equal or greater than 670 nm) [15], or live-imaged in a Nikon E200 equipped with a DS-Fi1 camera and Digital Sight software. Flow cytometry data were analysed using FlowJo 6.3 software (Tree Star, Inc.).

### Yeast assays

#### Strains

The DY1457 *S. cerevisiae* strains used were in the W303 background (W303 *MAT_ura3-52 leu2-3*,*112 trp1-1 his3-11 ade6 can1-100*). The *hem1Δ*(6D) strain (*hem1::LEU2trp1-1 his3-11 ura3-52 can1-100*) was constructed as described elsewhere [23]. Cells were maintained in YPD or appropriate synthetic complete (SC) media supplemented with 250 µM ALA (Frontier Scientific) [38].

#### Plasmid construction

*CeHRG-4* and *LHR1* (and HA-tagged versions) were cloned into the yeast vector pYes-DEST52 using *Bam*HI and *Xba*I sites. The plasmid pYes-DEST52 was used as vector control.

#### Spot growth assay

The plasmids were transformed into the strain *hem1Δ*(6D) using the lithium acetate method [39]. Transformants were selected on 2% w/v glucose SC (-Ura) plates supplemented with 250 µM ALA. Five or six colonies of each transformation were picked and restreaked on 2% w/v raffinose SC (-Ura) plates supplemented with 250 µM ALA to deplete glucose for 48 h. Prior to spotting, cells were cultivated in 2% w/v raffinose SC (-Ura) medium for 18 h to deplete hemin. Cells were then suspended in water to an OD_600_ of 0.2 (A_600_). 10 µl of ten-fold serial dilutions of each transformant were spotted onto 2% w/v raffinose SC (-Ura) plates supplemented with either 0.4% w/v glucose and 250 µM ALA (positive control), or 0.4% w/v galactose and various concentrations of hemin (no hemin addition as negative control), and incubated at 30°C for 3 days before imaging.

#### β-galactosidase reporter assay

The plasmids for *CeHRG-4* or *LHR1* expression were co-transformed into the strain *hem1Δ*(6D) with pCYC1-LacZ. Transformant selection was performed as described above using SC auxotrophic medium supplemented with 250 µM ALA. Cells were depleted for hemin in 2% w/v raffinose SC (-Ura, -Trp) medium for 12 h, and then were suspended in 10 ml 2% w/v raffinose SC (-Ura, -Trp) medium supplemented with 0.4% w/v galactose, and various concentration of hemin to an OD_600_ of 0.1. Cells were cultivated at 30°C, 225 rpm for 12 h and subjected to the β-galactosidase assay as described elsewhere [35].

#### ^55^Fe-heme uptake

Heme uptake in yeast was measured as described [24] with the following modifications. *S. cerevisiae* strain W303 was transformed with pYesDEST52 (vector) or pyLHR1 (yLHR1). Transformants were grown in synthetic defined medium with 2% raffinose and 0.4% galactose was added to induce expression of yLHR1. Cells were harvested, washed, and resuspended in uptake buffer (phosphate-buffered saline, 5% glucose, 0.5% tween 20, 0.5% bovine serum albumin) at an A_600_ of 4.0. After pre-incubation at 30°C for 30 min., [^55^Fe] hemin [40] was added at 1.2 µM and cells were incubated at 30°C for the indicated times. Cells were washed in assay buffer without glucose and retained [^55^Fe] hemin was measured by scintillation counting.
